# The Immunophilin-Like Protein XAP2 Is a Negative Regulator of Estrogen Signaling through Interaction with Estrogen Receptor α

**DOI:** 10.1371/journal.pone.0025201

**Published:** 2011-10-03

**Authors:** Wen Cai, Tatiana V. Kramarova, Petra Berg, Marta Korbonits, Ingemar Pongratz

**Affiliations:** 1 Department of Biosciences and Nutrition, Karolinska Institutet, Huddinge, Sweden; 2 Endocrinology, Barts and the London School of Medicine, Queen Mary University of London, London, United Kingdom; Oklahoma Medical Research Foundation, United States of America

## Abstract

XAP2 (also known as aryl hydrocarbon receptor interacting protein, AIP) is originally identified as a negative regulator of the hepatitis B virus X-associated protein. Recent studies have expanded the range of XAP2 client proteins to include the nuclear receptor family of transcription factors. In this study, we show that XAP2 is recruited to the promoter of ERα regulated genes like the breast cancer marker gene pS2 or GREB1 and negatively regulate the expression of these genes in MCF-7 cells. Interestingly, we show that XAP2 downregulates the E_2_-dependent transcriptional activation in an estrogen receptor (ER) isoform-specific manner: XAP2 inhibits ERα but not ERβ-mediated transcription. Thus, knockdown of intracellular XAP2 levels leads to increased ERα activity. XAP2 proteins, carrying mutations in their primary structures, loose the ability of interacting with ERα and can no longer regulate ER target gene transcription. Taken together, this study shows that XAP2 exerts a negative effect on ERα transcriptional activity and may thus prevent ERα-dependent events.

## Introduction

The Hepatitis B virus X protein associated protein 2 (XAP2) is a 37 kD immunophilin-like factor also known as aryl hydrocarbon receptor-associated protein 9 (ARA9) or aryl hydrocarbon receptor-interacting protein (AIP) [1], [2], [3]. XAP2 is an ubiquitously expressed protein, however, the intracellular levels of XAP2 vary considerably between different tissues, with high levels of expression observed in the spleen thymus and pituitary and low expression levels in the liver, kidney and lung [1] [4], [5], [6].

XAP2 is originally identified as a negative regulator of the hepatitis B virus X-associated protein [5]. Later, XAP2 was identified as an Hsp90-associated protein that specifically interacts with the aryl hydrocarbon receptor (AhR) and regulates both AhR intracellular localization [7] and protein stability by inhibiting AhR ubiquitination [8], [9], [10]. Additional studies, however, have expanded the range of XAP2 client proteins to include also signal transduction proteins like Ga13 [11] and nuclear receptor (NR) superfamily of transcription factors like GR [12], TRβ1 [13] and PPARα [14].

Estrogen receptor α (ERα) and β (ERβ) belong to the NR family and mediate the biological effects of estrogens [15]. In the absence of ligands the ERs are present in an inactive form [16]. Ligand-binding induces the recruitment of ER to estrogen response element (ERE) located within regulatory sequences of estrogen-responsive genes, resulting in the transcription activation of estrogen target genes. Estrogen signaling is involved in variety of physiological processes, both in females and males, in both reproductive and non-reproductive tissues [17], [18]. Although both ERα and ERβ are the mediators of the effects of estrogen, they have distinct, or even opposing effects in certain tissues where the biological action of estrogen ligands depends on a balance between ERα and ERβ [19], [20]. Several studies have demonstrated that the tumorigenic effects of estrogens are primarily mediated by ERα. Lifetime exposure and high estrogen levels and thus high ER transcriptional activity represent a risk factor for developing tumors in breast [21], endometrial [22], ovarian [23] pituitary [24] and thyroid tissues [25]. In contrast, ERβ has been shown to possess a tumor suppressive effect in tissues such as the prostate [26] and colon [27].

Recent studies suggest the involvement of XAP2 in a wide range of biological processes with tumorigenic outcome [28]. For example, disruption of XAP2 is observed in patients with family history of pituitary tumors [6], [29]. However, the mechanisms behind the tumor suppressive-activity of XAP2 have not been clarified yet. One possibility is that the XAP2 interacts with regulatory factors and thus modulates pathways involved in tumor development as well as other pathological processes. Previous studies have also demonstrated a physical and functional role of XAP2 in regulation of NR superfamily members PPARα and TRβ1, providing the possibility that XAP2 could act as a regulator in NR activities [13], [14]. Interestingly, several studies have showed that estrogen could induce the formation and development of pituitary tumor [30], [31], suggesting the possible involvement of ER-regulated signaling pathways in pituitary tumor pathogenesis. In addition, precautious puberty in a one-year-old female XAP2 mutation carrier has been reported [32], possibly implying a modified ER signaling in XAP2 mutated individuals.

In this study we have analyzed the impact of XAP2 on E_2_-dependent transcriptional activation. We show that XAP2 negatively regulates the transcriptional activity of ER in an isoform-specific manner, by inhibiting ERα-mediated but not ERβ-mediated transcription. Our studies demonstrate that XAP2 action is dependent on the protein-protein interaction of XAP2 with ERα on the promoter of ER-target gene.

Taken together, our experiments demonstrate that XAP2 is a negative regulator of ERα transcriptional activity and thus expand the list of XAP2 client proteins to include ERα.

## Materials and Methods

### Recombinant plasmids

The vectors pSG5-ERα, pSG5-ERβ, pCMV5-βGal (β-gal) and the 3×ERE-luciferase and pS2-luciferase reporter construct have been described elsewhere [33], [34]. Human pSG5-hXAP2 [9] and XAP2 mutation constructs [6] have been described elsewhere. Details regarding construction of the different plasmid constructs are available from the authors upon request.

### Cell cultures and Reporter assays

HeLa cells [7] and MCF-7 cells [35] were maintained in Dulbecco's modified Eagle's medium (DMEM, Invitrogen, Carlsbad, CA, USA) containing 10% fetal calf serum (FCS, Invitogen), 2 mM L-glutamine and 1% antibiotic (penicillin/streptomycin (100 units/ml)). HC11 and HC11 3×ERE cells [36] were propagated in RPMI 1640 medium (Invitrogen) supplemented with 10% FCS, 2 mM L-glutamine, 1% gentamycin (Invitrogen), 5 µg/ml insulin (Sigma-Aldrich, St Louis, MO, USA) and 10 ng/ml EGF (Sigma-Aldrich).

For reporter assays, cells were plated into 12-well or 24-well plates 24 h before transfection and maintained at 37°C. When 80% confluent the cells were transfected with Lipofectamin or Lipofectamin Plus according to the manufacturer's instructions (Invitrogen). A β-gal plasmid was used as an internal transfection control in the reporter assays. After 4 h of transfection, the medium was exchanged with phenol red-free medium supplemented with 5% dextran-coated, charcoal-treated FCS (DCC-FCS), cells were treated with DMSO or 10 nM E_2_ for another 48 h before luciferase activities were determined using luciferase assay kit (BioThema, Dalarö, Sweden).

### Cell extracts and immunoblot assay

For immunoprecipitation and Whole Cell Extract (WCE) experiments, HC11 cells were seeded out on 15 cm dishes. 24 h before treatment and/or transfection, the HC11 culture medium was changed to phenol red-free medium supplemented with DCC-FCS. The cells were then treated with DMSO or 10 nM E_2_ for 1 h. Cells were washed twice with cold PBS, collected by centrifugation, and suspended in WCE buffer (0.4 M KCl, 20 mM Hepes pH 7.4, 1 mM DTT and 20% glycerol) supplemented with a protease inhibitor (Complete-Mini; Roche Diagnostics, Mannheim, Germany), 10 mM Na_2_MoO_4_ and 25 µM Mg132 (Sigma-Aldrich). The freeze-thaw cycle with the cell suspensions were repeated 4 times. Lysates were cleared by centrifugation. 150 µg of cellular proteins were used for analysing the WCE experiments. For immunoprecipitation experiments 200 µg of cellular protein was incubated with 10 mM Na_2_MoO_4_ and anti-ARA9 (XAP2) antibody (Novus Biologicals, Littleton, CO) at 4°C for 1.5 h. Immunocomplexes were precipitated by adding 30 µl of 50% slurry of protein-G-Sepharose (Amersham-Pharmacia Biotech, Buckinghamshire, UK) plus 0.05% BSA followed by incubation at 4°C under slow rotation for 1.5 h. After centrifugation the resulting pellet were washed four times with 500 µl PBS.

For immunoprecipitaion of XAP2 mutations, HeLa cells were seeded out on 6-well-plate. 24 h after transfection, cells were then treated with DMSO or 10 nM E_2_ for 1 h. Immunoprecipitaion assays were performed using Pierce classic IP kit (Thermo Scientific) according to the manufacturer's instructions.

Precipitated proteins and whole cell extracts were analyzed by 7.5 or 10% SDS PAGE and transferred to nitrocellulose membranes. The membranes were treated with 5% nonfat milk in PBS at 4°C over night followed by incubation with primary antibodies. The primary antibodies used are ERα (Santa-Cruz; dilution 1∶1000), mouse c-myc (Santa Cruz; dilution 1∶500) and β-actin (Sigma-Aldrich; dilution 1∶10 000) in blocking solution. Horseradish peroxidase-conjugated anti-mouse or anti-rabbit immunoglobulins (DakoCytomation, Glostrup, Denmark) were used as secondary antibody. Immunocomplexes were visualized after extensive washing in PBS-0.1% Tween-20 using enhanced chemiluminescence reagents (ECL plus) (Amersham Pharmacia Biotech) according to the manufacturer's recommendations.

### RNA interference (RNAi)

siRNA against mouse XAP2 (msiXAP2) has been described previously [13]. siRNA against human XAP2 (hsiXAP2) was designed using the HiPerformance Design Algorithm licensed from Novartis AG (Qiagen) targeted hXAP2 nucleotides 113–133 (5′ AAGGAGGATGGCGGATATCAT3′). An AllStar scrambled sequence (Scr) from Novartis AG (Qiagen) where used as control. The cells were transfected with a final concentration of 20 nM of siRNA against XAP2 or Scr using Lipofectamine or Lipofectamine LTX with Plus Reagent according to the manufacturer's instructions (Invitrogen). After 48 h, cells were treated with DMSO or 10 nM E2 for 1 h and harvested for Western blot and immunoprecipitation experiments; Real-time RT-PCR experiments were performed essentially as described previously [37] for reporter assay in HC11 3×ERE cells.

### In vitro translation assay

In vitro translation was performed using rabbit reticulocyte lysate system (TNT® T7/SP6 Coupled Reticulocyte Lysate System, Cat#L5020, Promega), according to manufacturer's insctructions. In brief, purified XAP2-pSG5, ERα-pSG5 vector constructs (1 µg) were added to translation mix containing ^35^S-Methionine (500 µCi, 10 mCi/ml, Perkin Elmer), T7 polymerase, rabbit reticulocyte lysate, amino acid mixture (minus methyonine), RNase inhibitor and incubated for 90 min at 30°C. Equal amounts of translated products were then mixed and incubated on ice for 60 min, with gentle shaking. After incubation, XAP2 antibodies (NB100–127, Novus Biologicals) or rabbit IgG (Santa-Cruz Biotech) were added to each mix and incubated on ice for 60 min with gentle shaking, then Sepharose A/G was added and immunoprecipitated complexes were washed 3× with PBS, eluted with SDS-loading buffer and analysed by SDS protein gel electrophoresis.

### Re-ChIP assays

Sequential chromatin immunoprecipitation (Re-ChIP) assays were performed essentially as described previously [34]. MCF-7 cells were grown on 15 cm plates to 90% confluence in phenol red-free DMEM supplemented with 5% DCC-FCS. After treating with DMSO or 10 nM E_2_ for 45 min, cells were cross-linked for 15 min with 1.5% formaldehyde. Chromatin was then sonicated and an aliquot of the sonicated soluble chromatin was taken and later used as input control. Antibodies used were XAP2 (Novus Biologicals, Littleton, CO) and ERα (Santa-Cruz, H-184). After the first precipitation, specific antibody-protein complexes were bound to Sepharose G Fast Flow (Amersham Biosciences), washed and eluted with 10 mM dithiothreitol, precipitated with second antibodies, washed and eluted in 0.1 M NaHCO_3_, 1% SDS. Cross-linking was reversed at 65°C overnight. Eluted DNA fragments were purified using QIAquick Spin Kit (Qiagen, Valencia, CA). PCR experiments were performed at standard conditions and the PCR-amplified fragments were analyzed on 2% agarose gels. Real-time RT-PCR was performed for quantification. Primers: pS2: (F-5′-CCGGCCATCTCTCACTATGAA-3′) (R-5′-CCTCCCGCCAGGGTAAATAC-3′); GREB1: (F-5′-AGCAGTGAAAAAAAGTGTGGCAACTGGG-3′) (R-5′-CGACCCACAGAAATGAAAAGGCAGCAAACT-3′);

## Results

### XAP2 has suppressive effects on ERα target gene expression

**Figure 1:**
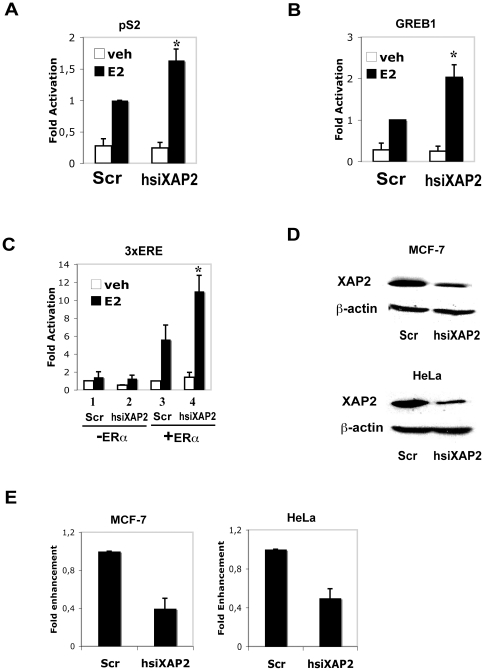
Suppressive effects of XAP2 on ERα-mediated gene expression. (A) The expression of endogenous pS2 gene in MCF-7 cells was monitored following the transfection with XAP2 siRNA (hsiXAP2) or scramble siRNA (Scr). After 48 h of transfection, cells were treated with DMSO (veh) or 10 nM E_2_ (E2) for 6 h before harvest. The mRNA levels of pS2 were determined by real-time RT-PCR and activity of scramble siRNA transfected E_2_ treated samples were arbitrarily set to 1. (B) The expression of endogenous GREB1 gene in MCF-7 cells was monitored following the transfection with XAP2 siRNA (hsiXAP2) or scramble siRNA (Scr). After 48 h of transfection, cells were treated with DMSO or 10 nM E_2_ for 6 h before harvest. The mRNA levels of GREB1 were determined by real-time RT-PCR and activity of scramble siRNA transfected E_2_ treated samples were arbitrarily set to 1. (C) ERα-independent (−ERα) or dependent (+ERα) activation of 3×ERE-TATA-Luc reporter gene transcription in HeLa cells was monitored following co-transfection with XAP2 siRNA (hsiXAP2) or scramble siRNA (Scr). After 4 h of transfection, cells were treated with DMSO or 10 nM E_2_ for 48 h before reporter gene activity was determined. Activity of scramble siRNA transfected DMSO treated cell samples were arbitrarily set to 1. (D) XAP2 and β-actin protein levels in MCF-7 and HeLa cells transfected with XAP2 siRNA (hsiXAP2) or Scramble siRNA (Scr) were determined by Western blot. (E) The protein expressions on Western blot in (D) were quantified by density of specific bands and normalized to β-actin. The XAP2/β-actin ratio in Scr sequence-transfected cells was arbitrarily set to 1. Data were expressed as means ± SE of three independent experiments performed in duplicate. *, *P*<0.05 (Student's *t* test).

Previous studies have shown a casual role for XAP2 as a tumor suppressor and possibly as a modulator of ER activity; however, the direct involvement between XAP2 and the ERs has not been assessed. Therefore, we decided to test whether XAP2 has any regulatory effect on ER-dependent gene expression. For this purpose, we performed siRNA experiments to knock down the intracellular levels of XAP2 in MCF-7 cells, a human breast adenocarcinoma cell line [35], extensively used to characterize E_2_ signaling pathways, and which expresses XAP2 and ERα (data not shown). We introduced siRNA constructs that targeted XAP2 mRNA or in control experiments, a scrambled sequence and assessed the effect of XAP2 knockdown on E_2_ target gene expression. Interestingly, when we knocked down XAP2 in MCF-7 cells (Fig. 1D–E, MCF-7), we observed a statistically significant up-regulation of endogenous expression of the breast cancer marker gene pS2 in the presence of E_2_ (Fig. 1A, compare Scr E_2_ and hsiXAP2 E_2_). In addition, the expression of another ERα-regulate gene GREB1 (growth regulation by estrogen in breast cancer 1) [38] is also increased upon XAP2 depletion in MCF-7 cells (Fig. 1B, compare Scr E_2_ and hsiXAP2 E_2_), suggesting a suppressive effect of XAP2 on the expression of ERα target genes. To confirm this observation, and to validate the role of ERα, we performed the similar RNAi assay in HeLa cells, where ERs are not expressed. We transfected HeLa cells with siRNA against XAP2 (hsiXAP2) or a scrambled sequence as negative control together with ERα expression construct and a synthetic 3×ERE reporter construct derived from the vitellogenin xenopus promoter. Knockdown of XAP2 (Fig. 1D–E, HeLa) resulted in increased ERα-mediated 3×ERE activation (Fig. 1C, compare 3 E_2_ and 4 E_2_), in accordance with the results we obtained in MCF-7 cells. In addition, no effect of XAP2 depletion was observed on 3×ERE dependent transcription in the absence of transfected ERα (Fig. 1C, compare 1 E_2_ and 2 E_2_), suggesting that the effects of XAP2 are mediated by ERα. Taken together, these results suggest that XAP2 has an inhibitory effect on E_2_-induced transcription of ERα target genes.

### XAP2 represses E_2_ transcriptional activity in an isoform-specific manner

**Figure 2:**
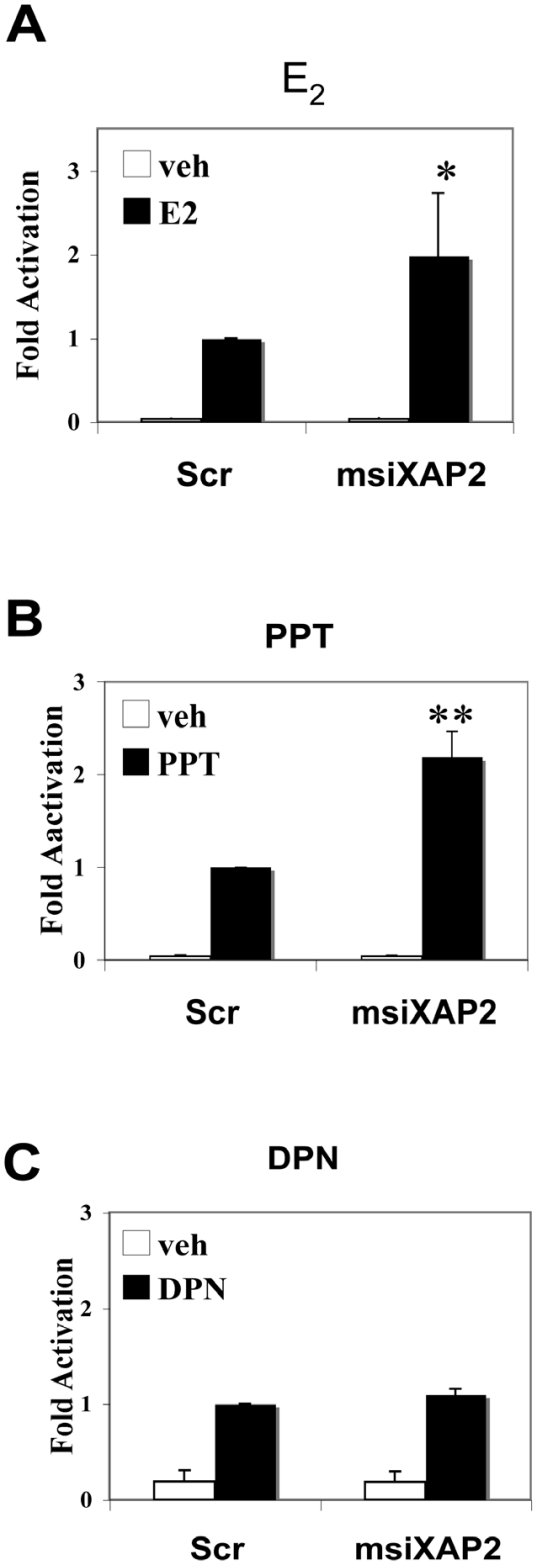
XAP2 knockdown has different influences on ERα- and ERβ-mediated ERE transcription. 3×ERE-HC11 cells were transfected with XAP2 siRNA (msiXAP2) or scrambled siRNA (Scr) sequences. Following transfection cells were incubated for 24 h and treated with (A) 10 nM E_2_, (B) 10 nM PPT (ERα selective ligand) or (C) 10 nM DPN (ERβ selective ligand) for another 24 h before the measurement of luciferase activity. Luciferase activity of cells transfected with Scr sequence in the presence of ligand was arbitrarily set to 1 and data expressed as means ± SD of three independent experiments performed in triplicate. *, *P*<0.05; **, *P*<0.01 (Student's *t* test).

Although both ERα and ERβ mediate estrogen signaling, biological functions of these two ER isoforms are distinct, especially in tumorogenesis [19], [20]. Therefore, we decided to investigate whether XAP2 has effects on both ERα and ERβ-dependent transcriptional regulation. For this purpose, we performed siRNA assay in stable HC11-3×ERE cells, a mouse mammary epithelial cell line, which expresses XAP2 (data not shown) and both estrogen receptor isoforms, ERα and ERβ [36]. HC11 cells were transfected with XAP2 siRNA (msiXAP2) or a scrambled sequence (Scr), and treated with different ER isoform-specific ligands, i.e. the ERα agonist propyl pyrazole triol (PPT) [39], the ERβ agonist diarylpropionitrile (DPN) [40] or the panagonist E_2_. Remarkably, after XAP2 depletion, no significant difference in 3×ERE luciferase activity was observed in DPN treated cells (Fig. 2C). However, luciferase expression was increased by 2 -fold in E_2_ treated cells (Fig. 2A) and 2.2 -fold in PPT treated cells (Fig. 2B), following XAP2 depletion. These experiments suggest that the influence XAP2 on ER transcriptional activity is ER isoform-specific.

**Figure 3:**
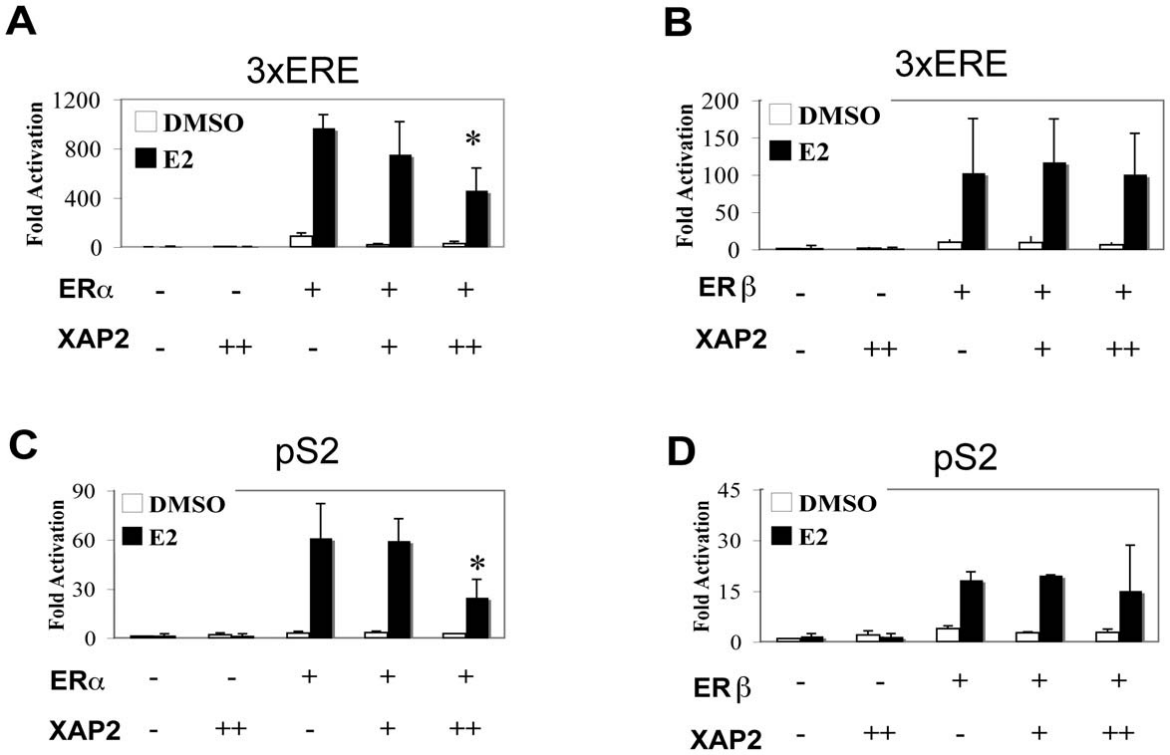
XAP2 represses ERα but not ERβ mediated transcription. (A–B) HeLa cells were transiently co-transfected with 1 ng of ERα (A) or ERβ (B) expression vectors together with increasing amounts of XAP2 (1–10 ng) together with 100 ng of a 3×ERE-TATA-Luc reporter. (C–D) HeLa cells were transiently transfected with 1 ng of ERα (C) or ERβ (D) expression vectors upon increasing amounts of XAP2 (1–10 ng) together with 100 ng of a pS2 promoter luciferase reporter construct. 3 h after transfection, cells were treated with DMSO or 10 nM E_2_ for 48 h. Whole cell extracts (WCE) were prepared and luciferase activity was measured. Reporter gene activity was determined and normalized to β-galactosidase. Results were compared to basic luciferase activity of the reporter constructs, which were arbitrarily set to 1. Data were expressed as means ± SD of three independent experiments performed in triplicate. *, *P*<0.05 (Student's *t* test).

To further verify that XAP2 modulates ERα-dependent but not ERβ-dependent E_2_ signaling, we performed transient transfections in HeLa cells, since this cell line expresses neither ERα nor ERβ, thus allowing us to assess the effects of XAP2 on the individual estrogen receptor isoforms. HeLa cells were transiently co-transfected with fixed amounts of ERα or ERβ expression vectors together with increasing amounts of XAP2. The effects of XAP2 on ERα and ERβ transcriptional activation was tested on two different estrogen-regulated luciferase reporter gene constructs, the 3×ERE reporter (Fig. 3A–B) and the promoter of pS2 gene (Fig. 3C–D). Following transfection, the cells were treated with 10 nM E_2_ or vehicle for 48 hours before the cells were harvested, and luciferase activity was determined as described previously [7]. Co-transfection with increasing amount of XAP2 expression vector resulted in significant dose-dependent reductions of ERα-mediated 3×ERE activity (Fig. 3A) as well as the pS2 promoter activity (Fig. 3C). However, co-transfection of ERβ and XAP2 did not result in any significant change of transcription induction of the reporter constructs (Fig. 3B and D).

Taken together, these results indicate that XAP2 negatively regulates E_2_-dependent transcriptional activity in an ER isoform-specific manner, by inhibiting ERα but not ERβ-mediated transcriptional activity.

### XAP2 interacts with ERα but has no effect on the intracellular level of ERα

**Figure 4:**
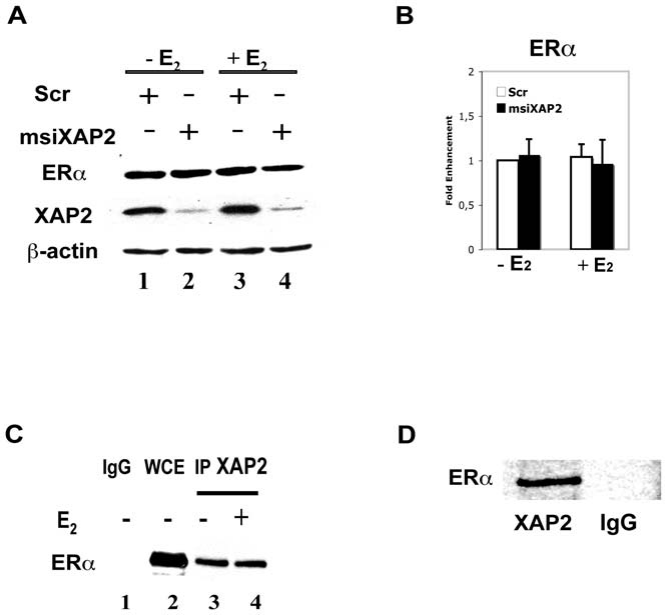
XAP2 interacts with ERα but has no effect on the intracellular level of ERα. (A) HC11 cells where transiently transfected with XAP2 siRNA (msiXAP2) (lanes 2,4) or a scrambled siRNA (Scr) (lanes 1,3). 48 h after transfection, cells were treated with DMSO (−E_2_) or 10 nM E_2_ (+E_2_) for 1 h before harvest. Whole cell extracts were prepared and Western blot experiments were performed with indicated antibodies; β-actin was used as a loading control. (B) The ERα protein levels shown in (A) were quantified by measuring the density of specific bands and normalizing to β-actin progein levels. The ERα/β-actin ratio in Scr (−E_2_) cells was arbitrarily set to 1 and data were expressed as means ± SE of three independent experiments. (C) HC11 cells were treated for 1 h with DMSO (−) or E_2_. Whole cell extract (WCE) was prepared and immunoprecipitation (IP) experiments were performed using a XAP2 antibody (lanes 3–4). The presence of ERα protein was monitored by Western blot analysis. WCE (lane 2) and an IgG antibody (lane 1) show the positive and negative controls, respectively. Data shown here is representative of three independent experiments. (D) Radioactively labeled proteins XAP2 and ERα synthesized by *in vitro* translation were mixed in equal amounts. After incubation (for details, see Materials and Methods), XAP2 antibodies or IgG antibodies were added to each protein mixture. Precipitated complexes were analyzed by SDS-PAGE. Data shown here is representative of three independent experiments.

Previous studies have shown that XAP2 stabilizes AhR protein levels by inhibiting protein degradation of the latent receptor [9]. To investigate whether alterations in ERα protein levels could account for the observed effect of XAP2 on ERα transcriptional activation, we performed siRNA assays in HC11 cells. We knocked down XAP2 levels in HC11 cells with siRNA (msiXAP2) and monitored ERα protein levels with Western blot. As shown in Fig. 4A, siRNA treatment led to a substantial reduction of XAP2 protein level (Fig. 4A, XAP2), however, this downregulation did not visibly influenced ERα protein levels (Fig. 4A–B, ERα), indicating that XAP2 has no direct effect on ERα protein stability. This observation suggests that the effects of XAP2 differ between ERα and the AhR, where XAP2 protects the non-activated, Hsp90-bound form of the AhR from ubiquitination [8], [9].

Given that XAP2 belongs to the family of TPR-containing protein and interacts with most of its partner proteins [28], we next determined whether XAP2 interacts with ERα. Co-immunoprecipitation experiments showed that XAP2 could interact with ERα (Fig. 4C) in HC11 cells, both in the absence and presence of E_2_. Furthermore, our *in vitro* translation assays again showed that XAP2 interacts with ERα (Fig. 4D).

Taken together, these data suggests that XAP2 interacts with ERα but has no effect on intracellular ERα level.

### XAP2/ERα interaction is crucial for XAP2 to inhibit ERα-mediated transcription

**Figure 5:**
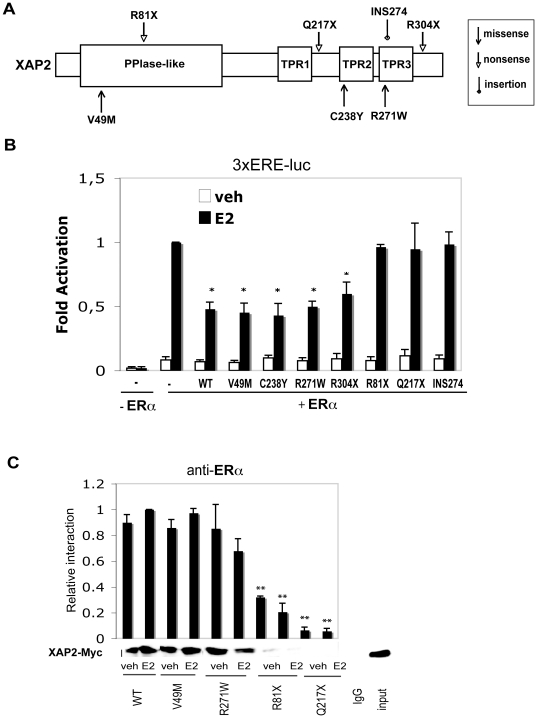
XAP2/ERα interaction is crucial for XAP2 to inhibit ERα-mediated transcription. (A) Schematic illustration of the XAP2 protein. The locations of the PPlase-like domain and the three TPRs are showed and mutation constructs used in this study are indicated. (B) HeLa cells were transiently co-transfected with expression vectors encoding indicated XAP2 mutations together with ERα and a 3×ERE-TATA-Luc reporter. 3 h after transfection, cells were treated with DMSO or 10 nM E_2_ for 48 h. Whole cell extracts (WCE) were prepared and luciferase activity was measured. Reporter gene activity was determined and normalized to β-galactosidase. Results were compared to luciferase activity of E_2_ dependent ERα-induced reporter activity, which were arbitrarily set to 1. (C) HeLa cells were transiently co-transfected with expression vectors encoding indicated cMyc-tagged XAP2 mutations together with ERα. 24 h after transfection, cells were treated with DMSO or 10 nM E_2_ for 1 h and immunoprecipitation (IP) experiments were performed using an ERα antibody. The presence of XAP2 proteins was monitored by Western blot analysis using c-Myc antibody. WCE (input) and an IgG antibody (IgG) show the positive and negative controls, respectively. XAP2 protein levels were quantified by measuring the density of specific bands. WT XAP2 transfected (+E_2_) cells was arbitrarily set to 1. Data were expressed as means ± SD of three independent experiments performed in triplicate. *, *P*<0.05 (Student's *t* test).

To better understand the mechanism by which XAP2 downregulats ERα transcriptional activity, we studied the effect of different XAP2 mutations on ERα-dependent transcription. For this purpose, we introduced plasmids encoding wild-type (WT) XAP2 and different XAP2 mutations into HeLa cells together with ERα and 3×ERE luciferase reporter using transient transfection experiments. XAP2 protein contains a peptidyl-prolyl cistrans isomerases (PPlase)-like domain and three tetratricopeptide (TPR) repeats. The three TPR domains each consist of two α-helices forming an antiparalell amphipathic structure that mediate intra- and inter-molecular interactions with many proteins [28]. The mutation constructs we examined are illustrated in Fig. 5A, they include three point mutations (V49M, C238Y, R271W), three nonsense mutations (R304X, R81X, Q217X) and an insertion mutation (INS274). As showed in Fig. 5B, mutations V49M, C238Y, R271W and R304X could still inhibit ERα-mediated 3×ERE transcription, whereas mutations R81X, Q217X and INS274 lost this ability.

Given that TPR domains could function as protein-protein interaction domains [41], we then examined whether the interaction of XAP2 with ERα is affected by mutations. Here we included WT XAP2, two point mutations (V49M, R271W) and two nonsense mutations (R81X, Q217X). We introduced the c-Myc-tagged XAP2 constructs into HeLa cells and performed co-immunoprecipitation (IP) assays. As shown in Fig. 5C, V49M and R271W were still able to interact with ERα, despite of the mutations; however, this interacting ability was significantly impaired for R81X and Q217X. Thus, the same mutated XAP2 proteins that loose the ability to mediate the downregulation of the ER-dependent transcription (Fig. 5B) also interacted less well with ERα (Fig. 5C). These results suggest that XAP2/ERα interaction is crucial for XAP2 to inhibit ERα-mediated transcription; it is also evident that the C-terminus of XAP2 protein (i.e. 2^nd^ and 3^rd^ TPR domains) seems to be important for mediating the XAP2-ERα protein-protein interactions.

### XAP2 is recruited to ER target gene promoters together with ERα and has inhibitory effects on the ERα recruitment

**Figure 6:**
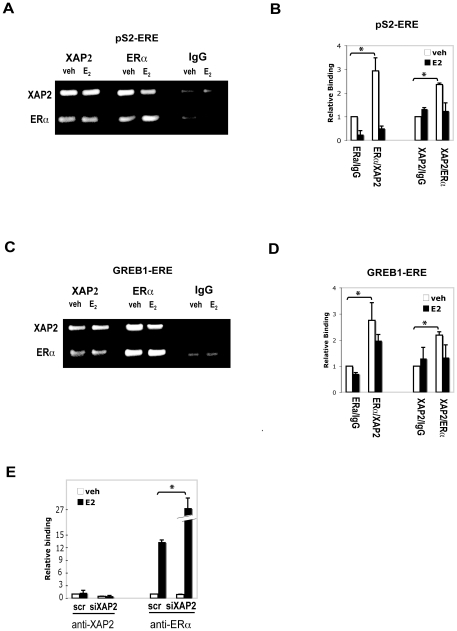
XAP2 is recruited to ER target gene promoters together with ERα and has inhibitory effects on the ERα recruitment. (A–D) Analysis of the ERα binding site on the ER target promoters with Re-ChIP assay. MCF-7 cells treated with DMSO (veh) or E_2_ were collected. Chromatin was first precipitated with the antibodies shown at the left side of the panel and DNA-protein complexes were precipitated again (re-ChIP) with the antibodies shown at the top of the panel. (A) Purified DNA fragments were subsequently analyzed by PCR with pS2-specific primers. Data shown here is representative of three independent experiments. (B) Real-time RT-PCR quantification of the re-ChIP assays with pS2-specific primers. IgG antibody-precipitated veh treated samples were arbitrarily set to 1. Data were expressed as means ± SE of two independent experiments performed in duplicate. (C) Purified DNA fragments were subsequently analyzed by PCR with GREB1-specific primers. Data shown here is representative of three independent experiments. (D) Real-time RT-PCR quantification of the re-ChIP assays with GREB1-specific primers. IgG antibody-precipitated veh treated samples were arbitrarily set to 1. Data were expressed as means ± SE of two independent experiments performed in duplicate. (E) Real-time RT-PCR analysis of the ERα binding site on the pS2 promoter with ChIP assay. MCF-7 cells were transfected with XAP2 siRNA (siXAP2) or a scrambled siRNA sequence (Scr) and treated with DMSO (veh) or E_2_. Cells were collected and chromatin was precipitated with the antibodies against XAP2 or ERα as shown. Purified DNA fragments were subsequently analyzed by real-time RT-PCR with pS2-specific primers. Scramble siRNA transfected veh treated samples were arbitrarily set to 1. Data were expressed as means ± SE of three independent experiments performed in duplicate. *, *P*<0.05 (Student's *t* test).

To monitor the possible presence of XAP2 on the regulatory promoter regions of ERα target genes, we performed sequential chromatin immunoprecipitation (Re-ChIP) assays. We treated MCF-7 cells with DMSO or 10 nM E_2_ and assessed recruitment of ERα and XAP2 to pS2 and GREB1 as described in Materials and Methods. As shown in Fig. 6A–B, in MCF-7 cells, XAP2 could be found on the pS2 promoter in form of XAP2-ERα complex; interestingly, E_2_ treatment leads to a considerably lower recruitment of the complexes to the promoter (Fig. 6A–B, E_2_), suggesting that the interaction of XAP2 with ERα on pS2 promoter is disrupted in the presence of E_2_. Similar results were observed on the ER binding region of GREB1 promoter (Fig. 6C–D).

In order to find out whether XAP2 has any effect on the recruitment of ERα to the target gene promoter, we performed the following procedure: intracellular levels of XAP2 protein in MCF-7 cells were reduced by siRNA (siXAP2) and ChIP assays were then carried out with antibodies against XAP2 or ERα. Upon siXAP2 treatment, the recruitment of XAP2 to the pS2 promoter was reduced as expected (Fig. 6E, anti-XAP2). However, the E_2_-dependent recruitment of both ERα to the pS2 promoter was increased after the XAP2 depletion (Fig. 6E, anti-ERα). Taken together, the data show that XAP2 is recruited to the ER binding region together with ERα and has an inhibitory effect on the ERα recruitment to the ER-binding region.

## Discussion

In the present study, we have investigated the role of XAP2 in regulation of E_2_-dependent transcriptional activation. XAP2 was originally identified as a negative regulator of the hepatitis B virus X-associated protein [5], and it has been shown to protect AhR from protein degradation by inhibiting AhR ubiquitination [9]. XAP2 is also known to be associated with a number of cellular factors, such as PPARα, TRβ1 and Gα13 protein [11], [13], [14]. Our current results demonstrate that XAP2 is involved in E_2_-mediated signaling pathway, interacting with ERα and reveal, for the first time, a mechanistic role of XAP2 affecting the transcription by regulating transcription factors on the target gene promoter.

**Figure 7:**
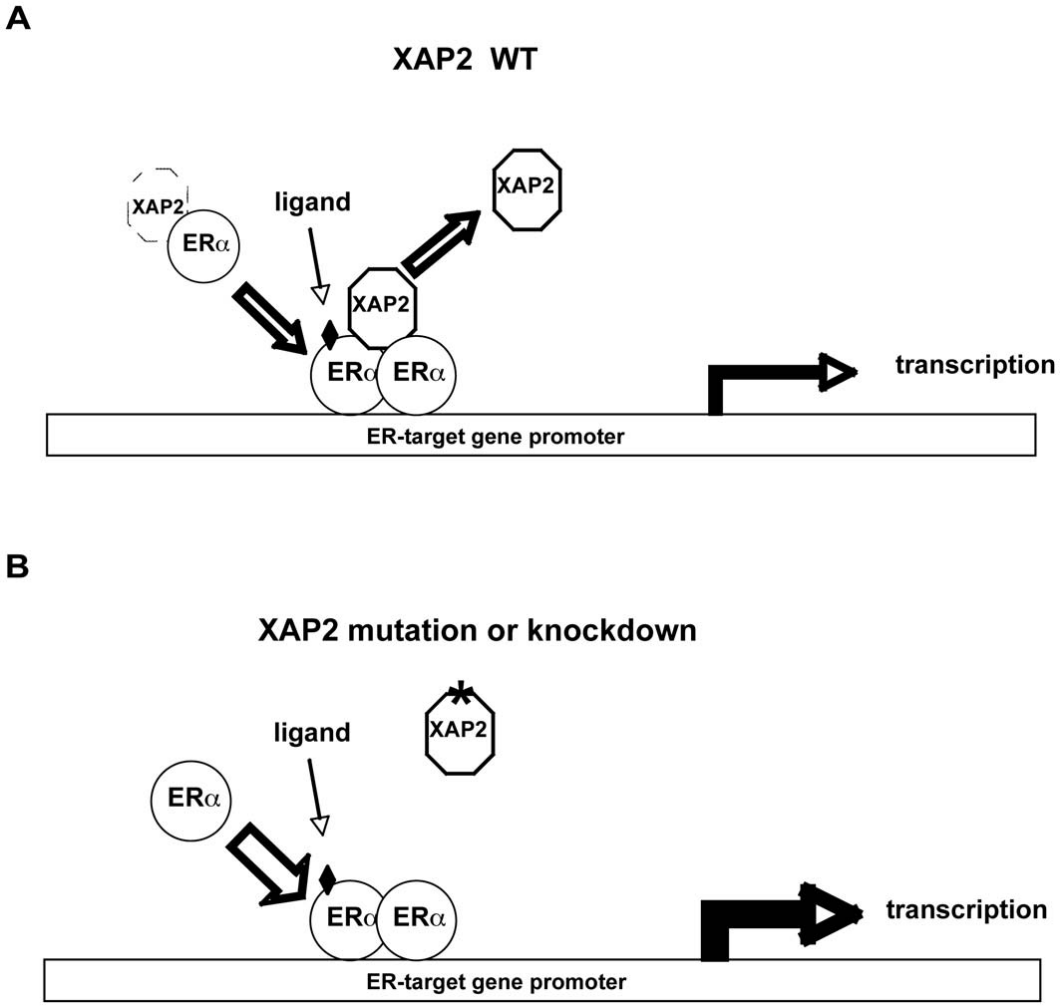
Schematic model of XAP2 regulation of ER-dependent transcription. (A) Upon the ligand (black diamond) binding, ERα is activated, WT XAP2 is starting to be released from the promoter whereas more ERα is recruited to the promoter and the target gene transcription is initiated. (B) When XAP2 is mutated or knocked down, there is less or no functional XAP2 that could interact with ERα, ERα is recruited more actively to the target gene promoter and the transcription is highly induced.

In MCF-7 cells, we observed a negative regulatory effect of XAP2 on the breast cancer marker gene pS2 as well as GREB1, another ER target gene (Fig. 1). Remarkably, our experiments show that XAP2 downregulates the E_2_-dependent transcriptional activation in an ER isoform-specific manner, by regulating ERα but not ERβ-mediated transcription (Fig. 2–3). Although XAP2 has previously been shown to protect AhR from protein degradation by inhibiting AhR ubiquitination [9], reduction of XAP2 protein does not affect the intracellular protein levels of ERα (Fig. 4A–B). Our results show that XAP2 could interact with ERα (Fig. 4C–D); using mutated forms of XAP2 protein we demonstrate that mutations that disrupt this interaction could no longer regulate ERα-mediated gene transcription (Fig. 5). We also show that XAP2 is recruited, or already present on ER-regulated promoters, together with ERα; addition of the ligand leads to a lower recruitment of the ERα/XAP2 complex (Fig. 6A–D). Knocking down XAP2 expression leads in its turn to increased recruitment of ERα to ER-target gene promoters (Fig. 6E). Based on these results, we propose a model showing the dynamic interaction of XAP2 with ERα in Fig. 7. In the absence of ligand, XAP2 is present on the ER-target gene promoter together with ERα and inhibits activity of ERα. Upon ligand binding, ERα is activated, XAP2 starts to be released from the promoter, as ERα is further recruited to the promoter and the target gene transcription is initiated (Fig. 7A). When XAP2 is mutated (as is found in tumors) or knocked down, there is less or no functional XAP2 that could interact with ERα, ERα is recruited more actively to the target gene promoter and the transcription is highly induced (Fig. 7B). Thus, XAP2 acts to inhibit or limit the degree of ERα transcriptional activity and is one of the key factors that affect the ER-mediated transcriptional regulation.

In this study, we propose that XAP2 represents candidate factor that influences ER-mediated transcription required for ligand inducibility of ERα, which may contribute to a cell-type or tissue-specific regulation of E_2_ responsive genes. In addition, XAP2 may serve as a negative regulator of numerous cellular regulatory pathways. Tissue distribution and intracellular levels of XAP2 may thereby affect the gene expression. Furthermore, the nature of ligands may also play an important role. E_2_ is the main estrogenic compound in pre-menopausal females, while the fetus and post-menopausal females rely on compounds like estriol and estrone respectively. These ligands are considerably weaker compared to E_2_ and this could partly be due to their ability to displace XAP2. In addition, xenoestrogens like bisphenol A or PCBs may also display different abilities to displace XAP2 and this may reflect on their endocrine disruptive effects. Clearly, additional studies need to be performed to better understand the full impact of XAP2 on nuclear receptor transcriptional regulation.

Germline mutations that disrupt the XAP2 protein have been reported in both familial and sporadic pituitary tumor patients and are possibly associated with other tumors [42]. In addition, targeted disruption of XAP2 in mice leads to cardiac malformation and embryonic lethality [43], [44]. These findings suggest XAP2 could be a key player in various physiological and pathological processes. In this study, we have demonstrated that XAP2 regulates the transcriptional response to E_2_ in an ER isoform-specific manner, as XAP2 inhibits ERα, but not ERβ-mediated transcription (Fig. 2–3). Thus, the disturbance in XAP2 expression, for instance, in individuals carrying XAP2 gene mutants, could over-activate ERα-mediated estrogen-signaling pathway. This will presumably break the normal balance between ERα and ERβ actions and may lead to a higher risk of developing estrogen-related disorders.

In conclusion, our study shows that XAP2 influences E_2_ signaling mediated by ERα by interacting with ERα. Further investigations, such as studies using clinical material from pituitary tumor patients with reported XAP2 mutations will allow to firmly establishing the physiological meaning of this regulation.
